# 4-{(*Z*)-(*sec*-Butyl­amino)(phen­yl)methyl­ene}-3-methyl-1-phenyl-1*H*-pyrazol-5(4*H*)-one

**DOI:** 10.1107/S160053680902950X

**Published:** 2009-08-08

**Authors:** Hai-Zhen Xu, Jian-Ping Xu, Yan-Wei Yuan, Jin Zhang, You-Quan Zhu

**Affiliations:** a College of Chemistry and Life Science, Tianjin Normal University, Weijin Road No. 241, Tianjin, People’s Republic of China; b Elementary Education College, Tianjin Normal University, Weijin Road No. 241, Tianjin, People’s Republic of China; c State Key Laboratory of Elemento-Organic Chemistry, Nankai University, Tianjin 300071, People’s Republic of China

## Abstract

In the title compound, C_21_H_23_N_3_O, the dihedral angles formed by the pyrazolone ring with two phenyl rings are 10.38 (8) and 76.94 (6)°. The *sec*-butyl­amino group is disordered over two positions, with refined site-occupancy factors of 0.730 (4) and 0.270 (4). The compound could potentially be ligand stabilized in the solid state in a keto–enamine tautomeric form. The amine functionality is involved in an intra­molecular N—H⋯O hydrogen bond, while weak inter­molecular C—H⋯O and C—H⋯N hydrogen bonds participate in the formation of the crystal structure.

## Related literature

For the anti­bacterial, biological and analgesic activity of metal complexes of 1-phenyl-3-methyl-4-benzoyl­pyrazolon-5-one, see: Li *et al.* (1997 ▶); Liu *et al.* (1980 ▶); Zhou *et al.* (1999 ▶).
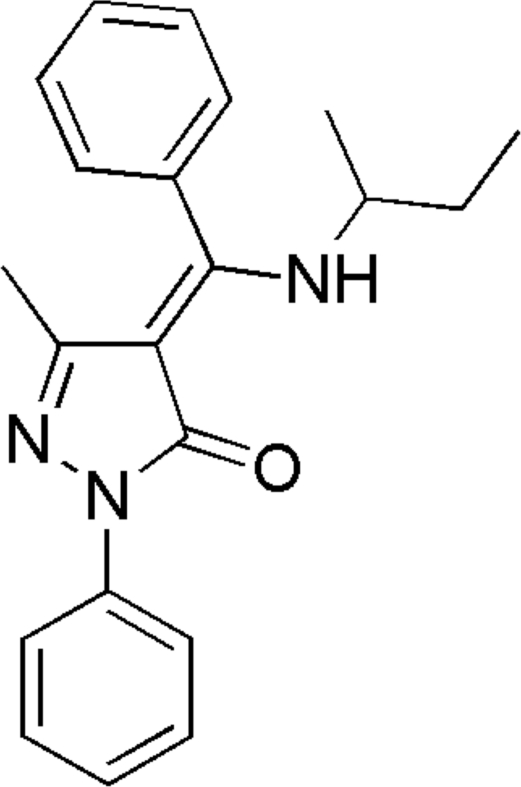


## Experimental

### 

#### Crystal data

C_21_H_23_N_3_O
*M* *_r_* = 333.42
Triclinic, 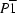

*a* = 9.3631 (19) Å
*b* = 10.077 (2) Å
*c* = 10.687 (2) Å
α = 107.07 (3)°
β = 100.30 (3)°
γ = 100.14 (3)°
*V* = 920.0 (4) Å^3^
*Z* = 2
Mo *K*α radiation
μ = 0.08 mm^−1^
*T* = 113 K
0.20 × 0.18 × 0.16 mm

#### Data collection

Rigaku Saturn CCD area-detector diffractometer
Absorption correction: multi-scan (*CrystalClear*; Rigaku, 2005 ▶) *T*_min_ = 0.985, *T*_max_ = 0.988
8309 measured reflections
4296 independent reflections
2944 reflections with *I* > 2σ(*I*)
*R*_int_ = 0.026

#### Refinement

*R*[*F* ^2^ > 2σ(*F* ^2^)] = 0.047
*wR*(*F* ^2^) = 0.135
*S* = 1.08
4296 reflections
272 parameters
16 restraints
H atoms treated by a mixture of independent and constrained refinement
Δρ_max_ = 0.32 e Å^−3^
Δρ_min_ = −0.21 e Å^−3^

### 

Data collection: *CrystalClear* (Rigaku, 2005 ▶); cell refinement: *CrystalClear*; data reduction: *CrystalClear*; program(s) used to solve structure: *SHELXS97* (Sheldrick, 2008 ▶); program(s) used to refine structure: *SHELXL97* (Sheldrick, 2008 ▶); molecular graphics: *SHELXTL* (Sheldrick, 2008 ▶); software used to prepare material for publication: *SHELXTL*.

## Supplementary Material

Crystal structure: contains datablocks global, I. DOI: 10.1107/S160053680902950X/bh2238sup1.cif

Structure factors: contains datablocks I. DOI: 10.1107/S160053680902950X/bh2238Isup2.hkl

Additional supplementary materials:  crystallographic information; 3D view; checkCIF report

**Fig. 1.**
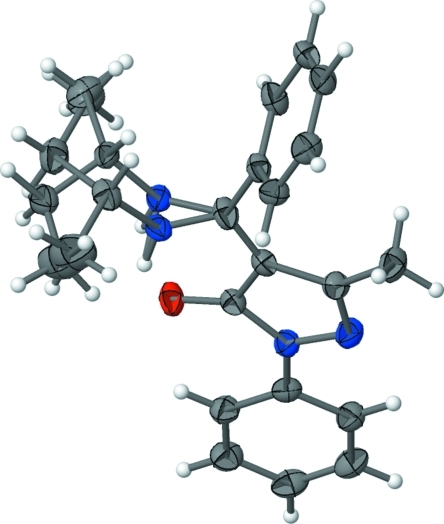
View of the title compound, with displacement ellipsoids drawn at the 30% probability level.

Enhanced figure: interactive version of Fig. 1

## Figures and Tables

**Table 1:** Selected bond lengths (Å)

O1—C7	1.2529 (17)
C7—C8	1.4382 (19)
C8—C11	1.402 (2)
C11—N3′	1.311 (5)
C11—N3	1.359 (2)

**Table 2:** Hydrogen-bond geometry (Å, °)

*D*—H⋯*A*	*D*—H	H⋯*A*	*D*⋯*A*	*D*—H⋯*A*
N3′—H3′⋯O1	0.904 (10)	1.99 (4)	2.705 (6)	135 (5)
N3—H3⋯O1	0.902 (10)	1.933 (15)	2.699 (2)	141.6 (18)
C16—H16*A*⋯O1^i^	0.95	2.53	3.2743 (19)	135
C13—H13*A*⋯N2^ii^	0.95	2.60	3.537 (2)	167

Symmetry codes: (i) 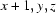; (ii) 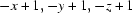.

**Fig. 2.**
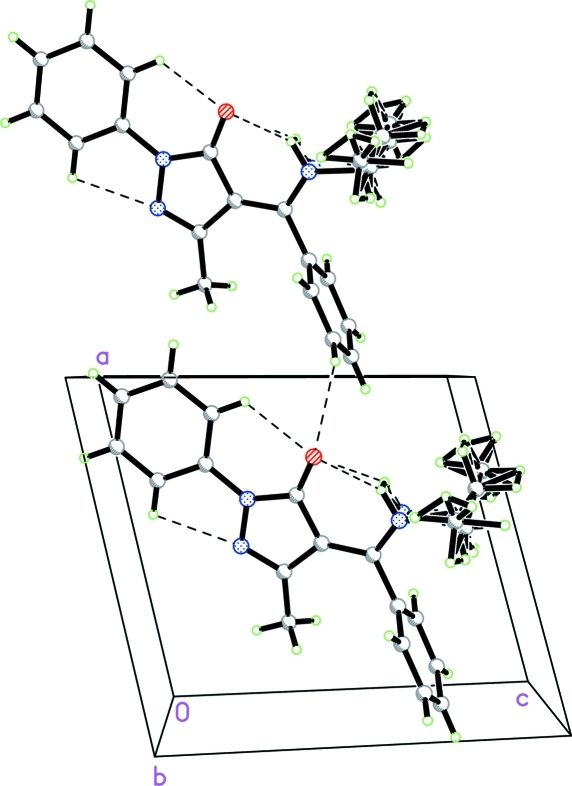
Intermolecular hydrogen bonds (dashed line) in the structure of (I).

## supplementary crystallographic information

### Comment

1-Phenyl-3-methyl-4-benzoylpyrazolon-5-one (HPMBP), an effective β-diketonate, is widely used and well known for its extractive ability. In recent years, HPMBP and its metal complexes have also been found to have good antibacterial and biological properties. Its metal complexes have analgesic activity (Liu *et al.*, 1980; Li *et al.*, 1997; Zhou *et al.*, 1999). In order to develop new medicines, we have synthesized the title compound, (I), and its structure is reported here.

The structure of (I) is shown in Fig. 1. The dihedral angles formed by the pyrazolone ring with the two phenyl rings C1···C6 and C12···C17 are 10.38 (8) and 76.94 (6)°, respectively. The O atom of the 3-methyl-1-phenylpyrazol-5-one moiety and the N atom of the *sec*-butylamino group are available for coordination with metals. The pyrazole ring is planar and atoms O1, C7, C8, C11 and N3 (or N3') are almost coplanar, the largest deviation being 0.0323 (13) Å [or 0.201 (3) Å] for atom C11. The dihedral angle between this mean plane and the pyrazoline ring of PMBP is 3.00 (11)° [or 12.10 (18)°]. The bond lengths within this part of the molecule (Table 1) lie between classical single- and double-bond lengths, indicating extensive conjugation. A strong intramolecular N3—H3···O1 hydrogen bond (Table 2) is observed, leading to a keto-enamine form. The molecule is further stabilized by C—H···O and C—H···N intramolecular hydrogen bonds (Table 2), while the crystal structure includes C—H···O and C—H···N intermolecular hydrogen bonds (Table 2 and Fig. 2).

### Experimental

Compound (I) was synthesized by refluxing a mixture of 1-phenyl-3- methyl-4-benzoylpyrazol-5-one (10 mmol) and *sec*-butylamine (10 mmol) in ethanol (80 ml) over a steam bath for about 4 h. Excess solvent was removed by evaporation and the solution was cooled to room temperature. After 2 days a yellow solid was obtained and this was dried in air. The product was recrystallized from ethanol, to afford yellow crystals of (I) suitable for X-ray analysis.

### Refinement

The *sec*-butylamino group shows positional disorder. At the final stage of the refinement, the occupancy factors of two possible sites, N3/C18/C19/C20/C21 and N3'/C18'/C19'/C20'/C21', converged to 0.730 (4) and 0.270 (4), respectively. The geometry of this disordered group was regularized using 16 restraints. C-bonded H atoms were positioned geometrically, with C—H = 0.95–1.00 Å and amine H atoms (H3 and H3') were found in a difference map. Amine H atoms were refined freely, while C-bonded H atoms were included in the final cycles of refinement using a riding model, with *U*_iso_(H) = 1.2*U*_eq_(CH_2_ and CH) or 1.5*U*_eq_(CH_3_).

### Crystal data

**Table:**

C_21_H_23_N_3_O	*Z* = 2
*M**_r_* = 333.42	*F*(000) = 356
Triclinic, *P*1	*D*_x_ = 1.204 Mg m^−^^3^
Hall symbol: -P 1	Mo *K*α radiation, λ = 0.71073 Å
*a* = 9.3631 (19) Å	Cell parameters from 2809 reflections
*b* = 10.077 (2) Å	θ = 2.2–27.9°
*c* = 10.687 (2) Å	µ = 0.08 mm^−^^1^
α = 107.07 (3)°	*T* = 113 K
β = 100.30 (3)°	Block, yellow
γ = 100.14 (3)°	0.20 × 0.18 × 0.16 mm
*V* = 920.0 (4) Å^3^	

### Data collection

**Table:**

Rigaku Saturn CCD area-detector diffractometer	4296 independent reflections
Radiation source: rotating anode	2944 reflections with *I* > 2σ(*I*)
confocal	*R*_int_ = 0.026
Detector resolution: 7.31 pixels mm^-1^	θ_max_ = 27.9°, θ_min_ = 2.2°
ω and φ scans	*h* = −12→12
Absorption correction: multi-scan (*CrystalClear*; Rigaku, 2005)	*k* = −11→13
*T*_min_ = 0.985, *T*_max_ = 0.988	*l* = −14→13
8309 measured reflections	

### Refinement

**Table:**

Refinement on *F*^2^	Secondary atom site location: difference Fourier map
Least-squares matrix: full	Hydrogen site location: inferred from neighbouring sites
*R*[*F*^2^ > 2σ(*F*^2^)] = 0.047	H atoms treated by a mixture of independent and constrained refinement
*wR*(*F*^2^) = 0.135	*w* = 1/[σ^2^(*F*_o_^2^) + (0.0708*P*)^2^ + 0.0483*P*] where *P* = (*F*_o_^2^ + 2*F*_c_^2^)/3
*S* = 1.08	(Δ/σ)_max_ = 0.005
4296 reflections	Δρ_max_ = 0.32 e Å^−^^3^
272 parameters	Δρ_min_ = −0.21 e Å^−^^3^
16 restraints	Extinction correction: *SHELXL97* (Sheldrick, 2008), Fc^*^=kFc[1+0.001xFc^2^λ^3^/sin(2θ)]^-1/4^
0 constraints	Extinction coefficient: 0.155 (17)
Primary atom site location: structure-invariant direct methods	

### Fractional atomic coordinates and isotropic or equivalent isotropic displacement parameters (Å^2^)

**Table:**

	*x*	*y*	*z*	*U*_iso_*/*U*_eq_	Occ. (<1)
O1	0.22643 (10)	0.11615 (12)	0.45573 (9)	0.0366 (3)	
N1	0.35369 (12)	0.31219 (12)	0.64961 (11)	0.0281 (3)	
N2	0.49444 (13)	0.40916 (13)	0.69807 (12)	0.0337 (3)	
C1	0.25255 (16)	0.31996 (14)	0.73383 (13)	0.0273 (3)	
C2	0.10503 (16)	0.24104 (16)	0.68465 (15)	0.0331 (3)	
H2A	0.0705	0.1794	0.5934	0.040*	
C3	0.00826 (17)	0.25261 (16)	0.76926 (16)	0.0377 (4)	
H3A	−0.0926	0.1982	0.7355	0.045*	
C4	0.05684 (19)	0.34266 (16)	0.90262 (16)	0.0385 (4)	
H4A	−0.0103	0.3510	0.9599	0.046*	
C5	0.20343 (19)	0.41966 (16)	0.95079 (15)	0.0387 (4)	
H5A	0.2377	0.4806	1.0423	0.046*	
C6	0.30202 (17)	0.40978 (15)	0.86797 (14)	0.0332 (3)	
H6A	0.4029	0.4640	0.9025	0.040*	
C7	0.33879 (15)	0.21606 (14)	0.52318 (13)	0.0261 (3)	
C8	0.47946 (14)	0.25477 (14)	0.49077 (13)	0.0260 (3)	
C9	0.56792 (16)	0.37451 (14)	0.60449 (14)	0.0293 (3)	
C10	0.72210 (17)	0.46179 (17)	0.62636 (16)	0.0402 (4)	
H10A	0.7509	0.5366	0.7153	0.060*	
H10B	0.7244	0.5061	0.5562	0.060*	
H10C	0.7924	0.4000	0.6220	0.060*	
C11	0.50824 (15)	0.17900 (16)	0.36892 (14)	0.0325 (4)	
C12	0.65777 (15)	0.20895 (15)	0.33908 (13)	0.0282 (3)	
C13	0.70609 (16)	0.32385 (15)	0.29807 (14)	0.0321 (3)	
H13A	0.6424	0.3850	0.2857	0.039*	
C14	0.84815 (17)	0.34901 (16)	0.27525 (15)	0.0359 (4)	
H14A	0.8823	0.4282	0.2481	0.043*	
C15	0.93991 (16)	0.25914 (16)	0.29191 (15)	0.0352 (4)	
H15A	1.0371	0.2769	0.2764	0.042*	
C16	0.89140 (16)	0.14361 (16)	0.33096 (15)	0.0354 (4)	
H16A	0.9546	0.0814	0.3411	0.042*	
C17	0.75037 (16)	0.11839 (16)	0.35532 (15)	0.0336 (4)	
H17A	0.7170	0.0394	0.3831	0.040*	
N3	0.4045 (2)	0.0595 (2)	0.2841 (2)	0.0280 (5)	0.730 (4)
H3	0.3183 (15)	0.043 (2)	0.310 (2)	0.033 (5)*	0.730 (4)
C18	0.4079 (7)	−0.1799 (5)	0.1378 (6)	0.0449 (11)	0.730 (4)
H18A	0.5018	−0.1793	0.1960	0.067*	0.730 (4)
H18B	0.4014	−0.2365	0.0442	0.067*	0.730 (4)
H18C	0.3232	−0.2222	0.1674	0.067*	0.730 (4)
C19	0.4035 (2)	−0.0265 (2)	0.14681 (18)	0.0280 (6)	0.730 (4)
H19	0.4940	0.0175	0.1222	0.034*	0.730 (4)
C20	0.2651 (2)	−0.0251 (3)	0.0494 (2)	0.0409 (7)	0.730 (4)
H20A	0.1751	−0.0664	0.0747	0.049*	0.730 (4)
H20B	0.2624	−0.0866	−0.0427	0.049*	0.730 (4)
C21	0.2595 (8)	0.1239 (7)	0.0476 (11)	0.0692 (17)	0.730 (4)
H21A	0.2495	0.1821	0.1354	0.104*	0.730 (4)
H21B	0.1735	0.1176	−0.0232	0.104*	0.730 (4)
H21C	0.3518	0.1684	0.0295	0.104*	0.730 (4)
N3'	0.3875 (6)	0.1202 (8)	0.2699 (5)	0.0319 (14)	0.270 (4)
H3'	0.300 (3)	0.126 (6)	0.294 (5)	0.033 (5)*	0.270 (4)
C18'	0.297 (3)	0.1380 (19)	0.049 (3)	0.0692 (17)	0.270 (4)
H18D	0.1966	0.1306	0.0662	0.104*	0.270 (4)
H18E	0.2874	0.0952	−0.0480	0.104*	0.270 (4)
H18F	0.3502	0.2390	0.0797	0.104*	0.270 (4)
C19'	0.3832 (6)	0.0589 (6)	0.1254 (4)	0.0332 (17)	0.270 (4)
H19'	0.4876	0.0723	0.1132	0.040*	0.270 (4)
C20'	0.3103 (7)	−0.0988 (6)	0.0803 (6)	0.0424 (19)	0.270 (4)
H20C	0.3058	−0.1420	−0.0168	0.051*	0.270 (4)
H20D	0.2063	−0.1109	0.0905	0.051*	0.270 (4)
C21'	0.391 (2)	−0.1790 (15)	0.1582 (19)	0.0449 (11)	0.270 (4)
H21D	0.4973	−0.1589	0.1573	0.067*	0.270 (4)
H21E	0.3467	−0.2820	0.1160	0.067*	0.270 (4)
H21F	0.3820	−0.1479	0.2517	0.067*	0.270 (4)

### Atomic displacement parameters (Å^2^)

**Table:**

	*U*^11^	*U*^22^	*U*^33^	*U*^12^	*U*^13^	*U*^23^
O1	0.0214 (5)	0.0490 (6)	0.0292 (5)	−0.0004 (4)	0.0063 (4)	0.0034 (4)
N1	0.0238 (6)	0.0280 (6)	0.0298 (6)	0.0032 (5)	0.0074 (5)	0.0070 (5)
N2	0.0286 (7)	0.0283 (6)	0.0382 (7)	0.0003 (5)	0.0107 (5)	0.0047 (5)
C1	0.0295 (7)	0.0259 (7)	0.0307 (7)	0.0102 (5)	0.0121 (6)	0.0109 (5)
C2	0.0309 (8)	0.0336 (8)	0.0338 (7)	0.0073 (6)	0.0117 (6)	0.0078 (6)
C3	0.0328 (8)	0.0373 (8)	0.0440 (8)	0.0071 (6)	0.0174 (7)	0.0114 (7)
C4	0.0463 (10)	0.0359 (8)	0.0423 (8)	0.0144 (7)	0.0254 (7)	0.0149 (7)
C5	0.0504 (10)	0.0354 (8)	0.0321 (7)	0.0123 (7)	0.0169 (7)	0.0088 (6)
C6	0.0346 (8)	0.0325 (8)	0.0320 (7)	0.0077 (6)	0.0093 (6)	0.0096 (6)
C7	0.0231 (7)	0.0311 (7)	0.0248 (6)	0.0068 (6)	0.0051 (5)	0.0105 (5)
C8	0.0216 (7)	0.0303 (7)	0.0272 (7)	0.0065 (5)	0.0054 (5)	0.0113 (6)
C9	0.0267 (7)	0.0266 (7)	0.0339 (7)	0.0047 (6)	0.0079 (6)	0.0099 (6)
C10	0.0342 (9)	0.0356 (8)	0.0400 (8)	−0.0042 (7)	0.0107 (7)	0.0031 (7)
C11	0.0228 (7)	0.0447 (9)	0.0275 (7)	0.0034 (6)	0.0054 (6)	0.0116 (6)
C12	0.0213 (7)	0.0356 (8)	0.0247 (6)	0.0028 (6)	0.0053 (5)	0.0082 (6)
C13	0.0306 (8)	0.0326 (7)	0.0357 (7)	0.0100 (6)	0.0108 (6)	0.0123 (6)
C14	0.0345 (8)	0.0323 (8)	0.0414 (8)	0.0028 (6)	0.0152 (7)	0.0127 (6)
C15	0.0206 (7)	0.0378 (8)	0.0409 (8)	0.0011 (6)	0.0092 (6)	0.0061 (6)
C16	0.0223 (7)	0.0378 (8)	0.0419 (8)	0.0082 (6)	0.0016 (6)	0.0105 (7)
C17	0.0274 (8)	0.0366 (8)	0.0353 (7)	0.0018 (6)	0.0023 (6)	0.0166 (6)
N3	0.0215 (10)	0.0311 (11)	0.0271 (9)	0.0031 (9)	0.0079 (7)	0.0040 (8)
C18	0.054 (2)	0.0336 (9)	0.048 (2)	0.0125 (9)	0.0204 (15)	0.0082 (11)
C19	0.0251 (11)	0.0284 (12)	0.0262 (10)	0.0039 (9)	0.0080 (8)	0.0032 (8)
C20	0.0368 (13)	0.0511 (15)	0.0311 (11)	0.0160 (11)	0.0051 (10)	0.0071 (10)
C21	0.096 (5)	0.0693 (18)	0.0589 (14)	0.044 (2)	0.019 (3)	0.0321 (16)
N3'	0.023 (3)	0.043 (4)	0.028 (3)	0.012 (3)	0.007 (2)	0.007 (3)
C18'	0.096 (5)	0.0693 (18)	0.0589 (14)	0.044 (2)	0.019 (3)	0.0321 (16)
C19'	0.025 (3)	0.046 (4)	0.031 (3)	0.006 (3)	0.011 (2)	0.015 (3)
C20'	0.035 (4)	0.043 (4)	0.039 (3)	0.000 (3)	0.008 (3)	0.007 (3)
C21'	0.054 (2)	0.0336 (9)	0.048 (2)	0.0125 (9)	0.0204 (15)	0.0082 (11)

### Geometric parameters (Å, °)

**Table:**

O1—C7	1.2529 (17)	C16—C17	1.386 (2)
N1—C7	1.3785 (18)	C16—H16A	0.9500
N1—N2	1.4019 (17)	C17—H17A	0.9500
N1—C1	1.4150 (18)	N3—C19	1.466 (3)
N2—C9	1.3119 (19)	N3—H3	0.902 (10)
C1—C2	1.387 (2)	N3—H3'	1.28 (4)
C1—C6	1.394 (2)	C18—C19	1.528 (4)
C2—C3	1.386 (2)	C18—H18A	0.9800
C2—H2A	0.9500	C18—H18B	0.9800
C3—C4	1.387 (2)	C18—H18C	0.9800
C3—H3A	0.9500	C19—C20	1.516 (3)
C4—C5	1.374 (2)	C19—H19	1.0000
C4—H4A	0.9500	C20—C21	1.517 (5)
C5—C6	1.385 (2)	C20—H20A	0.9900
C5—H5A	0.9500	C20—H20B	0.9900
C6—H6A	0.9500	C21—H21A	0.9800
C7—C8	1.4382 (19)	C21—H21B	0.9800
C8—C11	1.402 (2)	C21—H21C	0.9800
C8—C9	1.430 (2)	N3'—C19'	1.475 (6)
C9—C10	1.491 (2)	N3'—H3	1.149 (18)
C10—H10A	0.9800	N3'—H3'	0.904 (10)
C10—H10B	0.9800	C18'—C19'	1.527 (9)
C10—H10C	0.9800	C18'—H18D	0.9800
C11—N3'	1.311 (5)	C18'—H18E	0.9800
C11—N3	1.359 (2)	C18'—H18F	0.9800
C11—C12	1.4901 (19)	C19'—C20'	1.508 (7)
C12—C13	1.386 (2)	C19'—H19'	1.0000
C12—C17	1.390 (2)	C20'—C21'	1.519 (9)
C13—C14	1.388 (2)	C20'—H20C	0.9900
C13—H13A	0.9500	C20'—H20D	0.9900
C14—C15	1.380 (2)	C21'—H21D	0.9800
C14—H14A	0.9500	C21'—H21E	0.9800
C15—C16	1.379 (2)	C21'—H21F	0.9800
C15—H15A	0.9500		
			
C7—N1—N2	111.72 (11)	C15—C16—C17	119.92 (14)
C7—N1—C1	129.43 (12)	C15—C16—H16A	120.0
N2—N1—C1	118.78 (11)	C17—C16—H16A	120.0
C9—N2—N1	106.50 (11)	C16—C17—C12	119.82 (14)
C2—C1—C6	119.64 (14)	C16—C17—H17A	120.1
C2—C1—N1	121.12 (12)	C12—C17—H17A	120.1
C6—C1—N1	119.24 (13)	C11—N3—C19	128.01 (18)
C3—C2—C1	119.69 (14)	C11—N3—H3	114.1 (14)
C3—C2—H2A	120.2	C19—N3—H3	116.6 (13)
C1—C2—H2A	120.2	C11—N3—H3'	91 (2)
C2—C3—C4	120.83 (14)	C19—N3—H3'	116 (2)
C2—C3—H3A	119.6	H3—N3—H3'	47 (2)
C4—C3—H3A	119.6	N3—C19—C20	109.15 (17)
C5—C4—C3	119.10 (15)	N3—C19—C18	111.0 (3)
C5—C4—H4A	120.4	C20—C19—C18	110.6 (3)
C3—C4—H4A	120.4	N3—C19—H19	108.7
C4—C5—C6	121.02 (14)	C20—C19—H19	108.7
C4—C5—H5A	119.5	C18—C19—H19	108.7
C6—C5—H5A	119.5	C19—C20—C21	113.0 (3)
C5—C6—C1	119.71 (14)	C19—C20—H20A	109.0
C5—C6—H6A	120.1	C21—C20—H20A	109.0
C1—C6—H6A	120.1	C19—C20—H20B	109.0
O1—C7—N1	126.01 (13)	C21—C20—H20B	109.0
O1—C7—C8	129.17 (13)	H20A—C20—H20B	107.8
N1—C7—C8	104.80 (12)	C11—N3'—C19'	126.0 (5)
C11—C8—C9	132.65 (13)	C11—N3'—H3	102.0 (11)
C11—C8—C7	121.77 (13)	C19'—N3'—H3	113.7 (11)
C9—C8—C7	105.58 (12)	C11—N3'—H3'	115 (4)
N2—C9—C8	111.40 (13)	C19'—N3'—H3'	119 (4)
N2—C9—C10	118.92 (13)	H3—N3'—H3'	52 (4)
C8—C9—C10	129.65 (14)	C19'—C18'—H18D	109.5
C9—C10—H10A	109.5	C19'—C18'—H18E	109.5
C9—C10—H10B	109.5	H18D—C18'—H18E	109.5
H10A—C10—H10B	109.5	C19'—C18'—H18F	109.5
C9—C10—H10C	109.5	H18D—C18'—H18F	109.5
H10A—C10—H10C	109.5	H18E—C18'—H18F	109.5
H10B—C10—H10C	109.5	N3'—C19'—C20'	107.5 (5)
N3'—C11—C8	113.3 (3)	N3'—C19'—C18'	108.1 (14)
N3—C11—C8	118.82 (15)	C20'—C19'—C18'	112.7 (8)
N3'—C11—C12	120.1 (3)	N3'—C19'—H19'	109.5
N3—C11—C12	117.86 (15)	C20'—C19'—H19'	109.5
C8—C11—C12	122.46 (13)	C18'—C19'—H19'	109.5
C13—C12—C17	120.12 (13)	C19'—C20'—C21'	113.7 (7)
C13—C12—C11	122.04 (13)	C19'—C20'—H20C	108.8
C17—C12—C11	117.84 (13)	C21'—C20'—H20C	108.8
C12—C13—C14	119.60 (14)	C19'—C20'—H20D	108.8
C12—C13—H13A	120.2	C21'—C20'—H20D	108.8
C14—C13—H13A	120.2	H20C—C20'—H20D	107.7
C15—C14—C13	120.10 (14)	C20'—C21'—H21D	109.5
C15—C14—H14A	119.9	C20'—C21'—H21E	109.5
C13—C14—H14A	119.9	H21D—C21'—H21E	109.5
C16—C15—C14	120.43 (14)	C20'—C21'—H21F	109.5
C16—C15—H15A	119.8	H21D—C21'—H21F	109.5
C14—C15—H15A	119.8	H21E—C21'—H21F	109.5
			
C7—N1—N2—C9	−0.44 (16)	C7—C8—C11—N3	−5.0 (2)
C1—N1—N2—C9	176.80 (12)	C9—C8—C11—C12	4.8 (2)
C7—N1—C1—C2	−12.6 (2)	C7—C8—C11—C12	−174.22 (13)
N2—N1—C1—C2	170.69 (12)	N3'—C11—C12—C13	76.6 (4)
C7—N1—C1—C6	168.11 (13)	N3—C11—C12—C13	111.8 (2)
N2—N1—C1—C6	−8.57 (19)	C8—C11—C12—C13	−78.87 (19)
C6—C1—C2—C3	0.2 (2)	N3'—C11—C12—C17	−104.2 (4)
N1—C1—C2—C3	−179.08 (13)	N3—C11—C12—C17	−69.0 (2)
C1—C2—C3—C4	0.3 (2)	C8—C11—C12—C17	100.28 (17)
C2—C3—C4—C5	−0.7 (2)	C17—C12—C13—C14	−0.9 (2)
C3—C4—C5—C6	0.8 (2)	C11—C12—C13—C14	178.22 (13)
C4—C5—C6—C1	−0.3 (2)	C12—C13—C14—C15	0.7 (2)
C2—C1—C6—C5	−0.1 (2)	C13—C14—C15—C16	0.2 (2)
N1—C1—C6—C5	179.13 (13)	C14—C15—C16—C17	−0.8 (2)
N2—N1—C7—O1	178.94 (13)	C15—C16—C17—C12	0.6 (2)
C1—N1—C7—O1	2.1 (2)	C13—C12—C17—C16	0.3 (2)
N2—N1—C7—C8	0.35 (15)	C11—C12—C17—C16	−178.90 (13)
C1—N1—C7—C8	−176.52 (12)	N3'—C11—N3—C19	85.3 (6)
O1—C7—C8—C11	0.6 (2)	C8—C11—N3—C19	172.81 (19)
N1—C7—C8—C11	179.14 (12)	C12—C11—N3—C19	−17.5 (3)
O1—C7—C8—C9	−178.66 (14)	C11—N3—C19—C20	−118.9 (3)
N1—C7—C8—C9	−0.13 (14)	C11—N3—C19—C18	118.9 (4)
N1—N2—C9—C8	0.35 (16)	N3—C19—C20—C21	61.1 (5)
N1—N2—C9—C10	178.68 (12)	C18—C19—C20—C21	−176.5 (5)
C11—C8—C9—N2	−179.29 (15)	N3—C11—N3'—C19'	−85.2 (8)
C7—C8—C9—N2	−0.14 (16)	C8—C11—N3'—C19'	167.1 (5)
C11—C8—C9—C10	2.6 (3)	C12—C11—N3'—C19'	9.5 (9)
C7—C8—C9—C10	−178.24 (15)	C11—N3'—C19'—C20'	116.6 (7)
C9—C8—C11—N3'	−152.2 (4)	C11—N3'—C19'—C18'	−121.5 (11)
C7—C8—C11—N3'	28.8 (4)	N3'—C19'—C20'—C21'	−58.9 (10)
C9—C8—C11—N3	174.01 (18)	C18'—C19'—C20'—C21'	−177.9 (17)

### Hydrogen-bond geometry (Å, °)

**Table:**

*D*—H···*A*	*D*—H	H···*A*	*D*···*A*	*D*—H···*A*
N3'—H3'···O1	0.90 (1)	1.99 (4)	2.705 (6)	135 (5)
N3—H3···O1	0.90 (1)	1.93 (2)	2.699 (2)	142 (2)
C2—H2A···O1	0.95	2.29	2.9243 (19)	123
C6—H6A···N2	0.95	2.44	2.777 (2)	101
C16—H16A···O1^i^	0.95	2.53	3.2743 (19)	135
C13—H13A···N2^ii^	0.95	2.60	3.537 (2)	167

Symmetry codes: (i) *x*+1, *y*, *z*; (ii) −*x*+1, −*y*+1, −*z*+1.
